# Relationship between sarcopenia/paravertebral muscles and the incidence of vertebral refractures following percutaneous kyphoplasty: a retrospective study

**DOI:** 10.1186/s12891-022-05832-6

**Published:** 2022-09-22

**Authors:** Qi Chen, Chenyang Lei, Tingxiao Zhao, Zhanqiu Dai, Jun Zhang, Yongming Jin, Chen Xia

**Affiliations:** 1 Department of Orthopedic Surgery, Zhejiang Provincial People’s Hospital, People’s Hospital of Hangzhou Medical College, Hangzhou, 310014 Zhejiang People’s Republic of China; 2 Tongde Hospital of Zhejiang Province, Hangzhou, China; 3 Spine Lab, Department of Orthopedic Surgery, The First Affifiliated Hospital, Medical College of Zhejiang University, Hangzhou, China

**Keywords:** Sarcopenia, Paravertebral muscles, Osteoporotic vertebral compression, Elderly

## Abstract

**Background:**

This study aimed to reveal the associations of osteoporotic vertebral compression refracture (OVCRF) incidence with sarcopenia and paravertebral muscles (PVM).

**Methods:**

A total of 214 elderly patients who underwent percutaneous kyphoplasty in our hospital between January 2017 and December 2019 were analyzed. Data on possible risk factors, including sex, age, weight, height, diabetes, treated vertebral levels (thoracolumbar junction [(T10–L2]), vacuum clefts, and body mass index (BMI), were collected. Preoperative bone mineral density (BMD) and appendicular muscle mass were evaluated using dual-energy X-ray absorptiometry. Nutritional status was evaluated using the Mini Nutritional Assessment. Magnetic resonance imaging was performed to evaluate the physiological cross-sectional area of the PVM.

**Results:**

Overall, 74 (15 men and 59 women) and 60 (55 women and 14 men) patients developed OVCRF and sarcopenia, respectively. Sarcopenia is related to advanced age, ower BMD and BMI values. Sarcopenia-related indicators (PVM fat rate, appendicular muscle mass index, grip strength) were significantly lower in the sarcopenia group. Univariate analysis showed a correlation between OVCRF and BMD, BMI, diabetes, sarcopenia, and age. Multivariate analysis suggested that fatty infiltration of the PVM, BMD, sarcopenia, diabetes, BMI, and treated vertebral level remained as the independent predictors of OVCRF (*p* < 0.05).

**Conclusions:**

The association between sarcopenia and PVM as independent risk factors for OVCRF was established in this study; therefore, sarcopenia should be greatly considered in OVCRF prevention.

**Supplementary Information:**

The online version contains supplementary material available at 10.1186/s12891-022-05832-6.

## Background

With the increasing aging population, the incidence of osteoporotic vertebral compression fracture (OVCF) in the elderly is also rising, thereby leading to lower back pain and a bedridden status [1]. OVCF seriously affects the quality of life of patients because of its high morbidity and mortality risk [2]. After evaluation, OVCF are usually treated conservatively with analgesics, appropriate bed rest, braces, and rehabilitation therapy [3]. However, this conservative treatment is only effective in two-thirds of patients, and the remaining patients still have persistent pain, limited mobility, and even neurological impairment after conservative treatment [4, 5]. With the development of minimally invasive spine surgery techniques over the past two decades, percutaneous vertebroplasty (PVP) and percutaneous kyphoplasty (PKP) have been widely used to treat acute OVCF [6]. After surgery, most patients experience good pain relief and improve quality of life [7]; However, some patients experience pain again after initial surgery and standard anti-osteoporosis therapy, and A new fracture of the vertebral body will be found after the examination [8, 9]. Previous research has found there is a risk of adjacent segment fractures after PKP and PVP operations. Different literatures suggest that the incidences of postoperative refractures for both are as follows: PVP (8–52%), PKP (3–29%) [10, 11]. Previous studies have analyzed risk factors for refractures, including osteoporosis, gender, steroid use, and vacuum fissures [12].

Sarcopenia is a syndrome characterized by a decline in skeletal muscle mass and low muscle strength and physical performance due to aging [13]. According to the Asian Working Group for Sarcopenia (AWGS) criteria, the estimated prevalence of sarcopenia among the general older population ranges from 4.1% to 11.5% [14]. It has been reported that sarcopenia poses a heavy economic burden on society [15]. The bones and muscles are connected to each other by their adjacent surfaces as well as chemical and metabolic properties [16]. Previous studies have shown that sarcopenia is independently associated with osteoporosis [17]. Osteoporosis and sarcopenia often occur concurrently and lead to an increased risk of fragility fractures in aging populations [18]. The paravertebral muscles (PVM) play an important role in maintaining the stability of the spine [19, 20]. Movement of the vertebrae requires the PVM to contract and work against the levers of an internal skeleton. Several studies have reported that a decrease in PVM is strongly associated with increased lower back pain [21, 22]. In sarcopenia, PVM atrophy (PMA) may decrease the stability of the spine activity and increase the vertebral body stress, thus leading to OVCF. Although several studies have investigated the relationship between sarcopenia and OVCF [23, 24], few studies have considered the important role of sarcopenia in vertebral refracture after percutaneous procedures. The present study aimed to evaluate the relationship of sarcopenia and PVM with the occurrence of osteoporotic vertebral compression refracture (OVCRF) following PKP and to determine possible major risk factors associated with new compression fractures.

## Methods

### Patients

**Fig. 1:**
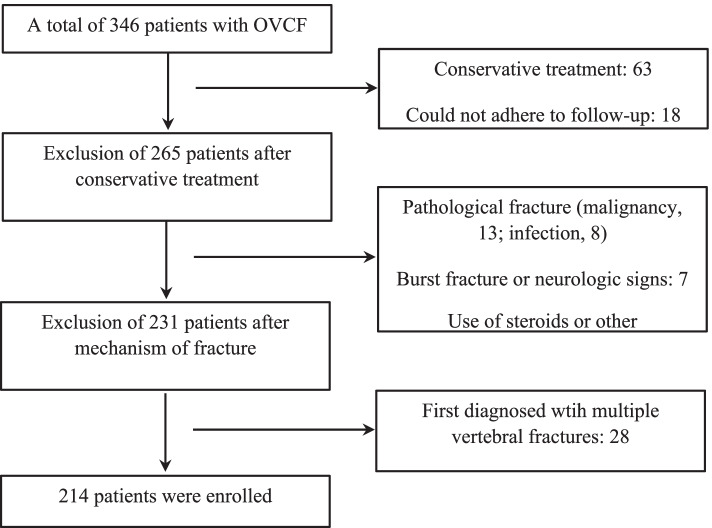
Trial profile

This retrospective study included consecutive patients who underwent PKP at the Department of Orthopedic Surgery, Zhejiang Provincial People’s Hospital, between January 2017 and December 2019. A total of 346 patients were screened; those with (i) Chinese ethnicity, (ii) a record of preoperative plain radiography and magnetic resonance imaging (MRI), (iii) PKP treatment of first-ever acute or subacute single-level vertebral compression fracture, (iv) The patient has no postoperative complications (anaphylactic shock, infiltration of bone cement into the spinal canal, or postoperative neurological deficit), (v) The patient has no history of other trauma after surgery (vi) Regular visits to the hospital for regular imaging examinations receive standard anti-osteoporosis treatment within 1 year were included in the study.Those with (i) pathological fractures caused by malignancy, infection or other diseases (ii) fractures with neurological symptoms, (iii) multiple vertebral fractures, (iv) patients with serious diseases who cannot be regularly visited for follow-up, (v) patients with severe liver and kidney disease and (vi) patients using steroid drugs were excluded. After applying the inclusion and exclusion criteria, 132 patients were excluded, and 214 patients were finally enrolled in the study (Fig. 1).

### Follow-up strategy

**Fig. 2:**
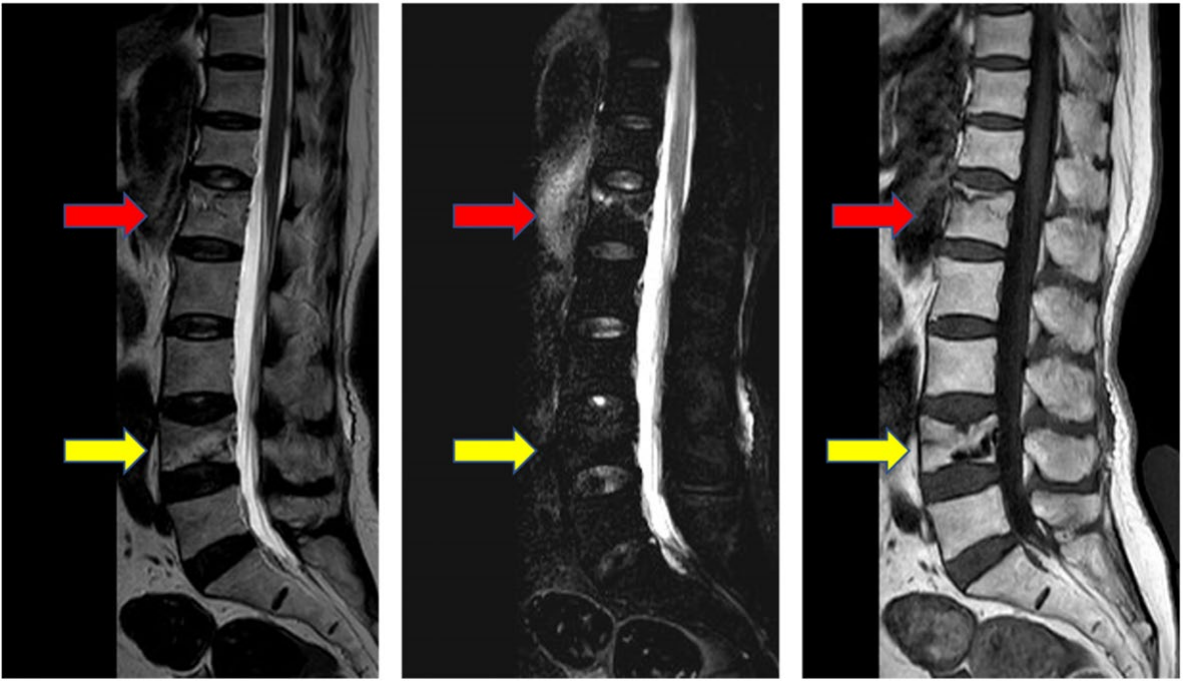
MRI example of vertebral refracture. Magnetic resonance imaging showed an old L4 fracture and a recurrent fracture of the L1 vertebral body in an 84-year-old female

The patients we followed up came to the outpatient clinic every month for the first 3 months. All patients were prescribed anti-osteoporosis medications, including bisphosphonates, calcium supplementation, and vitamin D. Specifically, alendronate (70 mg/week), caltrate (600–1200 mg/day), and calcitriol (0.25–0.5 mg/day) were prescribed after enrollment. However, anti-osteoporosis medication was administered on a case-by-case basis. The patients were told to return to the hospital at any point when there was recurrent back pain. Whenever a vertebral refracture was suspected, the patient underwent MRI to identify a new fracture (Fig. 2), and these patients were enrolled in the OVCRF group. The other groups were considered the control group.

### Data collection

Baseline data of the elderly patients, including sex, age, weight, height, diabetes, and body mass index (BMI), were collected. Preoperative bone mineral density (BMD) was measured using dual-energy X-ray absorptiometry (DEXA). Nutritional status was evaluated using the Mini Nutritional Assessment (MNA). The sum of the MNA score distinguishes between elderly patients with (i) adequate nutritional status (MNA > 24), (ii) a risk for malnutrition (MNA 17–23.5) and (iii) protein calorie undernutrition (MNA < 17). We also collected information on other high-risk factors, including treated vertebral levels (thoracolumbar junction [T10–L2]) and vacuum clefts.

### Evaluation of sarcopenia and PVM

According to the AWGS guidelines, three assessments are used to define sarcopenia: handgrip strength, physical performance, and appendicular muscle mass. Low handgrip strength was defined as ≤ 26 kg for men and ≤ 18 kg for women. Grip strength was measured three times on the dominant hand using an electronic hand dynamometer (GRIP-D5101, Takei, Niigata, Japan). The mean of these three measurements was used for the calculations. Physical performance was evaluated using the 6-m gait speed test, and low gait speed was defined as ≤ 0.8 m/s. An appendicular muscle mass index (AMI) cut-off value of 7.0 kg/m^2^ in men and 5.4 kg/m^2^ in women was also used. The appendicular muscle mass was evaluated by DEXA and converted to an AMI, which was calculated by dividing the absolute appendicular muscle mass by height in meters squared (kg/m^2^).

**Fig. 3:**
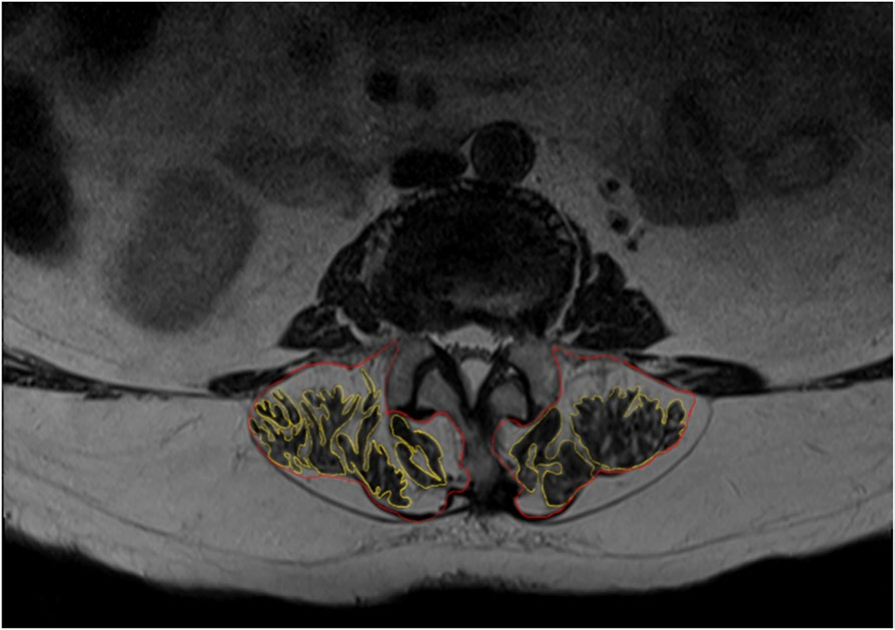
MRI performed to evaluate the physiological cross-sectional area of the PVM

MRI was performed to evaluate the physiological cross-sectional area (PCSA) of the PVM. The PCSA at the superior endplate of L3 was measured using a digital image processing software (Image J, National Institutes of Health, Bethesda, MD, USA). To determine the fatty infiltration of PVM, the region of interest was drawn around the target muscles on each side of the spine. Fats, bony structures, and other nearby soft tissues were not included. The rate of PMA = (PCSA — muscles of interest area) / (PCSA) × 100% (Fig. 3).

### Statistical analysis

Univariate analyses were conducted to evaluate the association between each risk factor and sarcopenia. In addition, univariate analysis was conducted to assess the relationships between the OVCRF and other variables. Descriptive statistics (for categorical risk factors, mean, and standard deviation for continuous risk factors) were also computed. All risk factors were entered into a multivariate logistic regression model, followed by a backward selection process based on the Akaike information criterion (AIC). The model with the lowest AIC value was chosen as the final model. Odds ratios (ORs) and 95% confidence intervals (CIs) were obtained for the risk factors in the final model. Statistical significance was set at *p* < 0.05. Data were analyzed using R software (version 4.1.2,).

## Results

### Patient demographic information

Table 1 shows the basic demographic information of the patients. This study included 59 men and 144 women with an average age of 77.98 ± 3.92 years. According to the sarcopenia diagnostic criteria, 69/214 patients (55 women and 14 men) had sarcopenia. Using a univariate logistic random effects model to analyze all variables, we found that patients with sarcopenia were older and had lower BMD and BMI values. In addition, three sarcopenia-related indicators (PVM fat rate, AMI, and grip strength) were significantly lower in the sarcopenia group.

**Table 1:** Basic patient data

Variable	Sarcopenia (*n* = 69)	Non-sarcopenia (*n* = 145)	*p*-value
Sex			
Male	20% (14)	32% (46)	0.08
Female	80% (55)	68% (99)	
Age (years)	80.76 ± 4.02	76.64 ± 5.69	< 0.001
Height (m)	1.56 ± 0.05	1.57 ± 0.05	0.12
Weight (kg)	45.74 ± 3.56	50.17 ± 4.01	< 0.001
BMI (kg/m^2^)	18.76 ± 1.19	20.27 ± 1.15	< 0.001
MNA			
A	3% (2)	18% (26)	< 0.001
B	32% (22)	45% (65)	
C	65% (45)	37% (54)	
BMD (T-score)	-3.01 ± 0.43	-2.2 ± 0.4	< 0.001
Diabetes			
Yes	41% (28)	12% (17)	< 0.001
No	59% (41)	88% (128)	
Vertebral refracture			
Yes	84% (58)	11% (16)	< 0.001
No	16% (11)	89% (129)	
AMI (kg/m^2^)	5.04 ± 0.68	6.78 ± 1.01	< 0.001
Handgrip strength (kg)	11.39 ± 1.12	12.67 ± 1.16	< 0.001
Rate of PMA (%)	56.30 ± 4.13	49.03 ± 4.32	< 0.001

### Risk factors for OVCRF

**Fig. 4:**
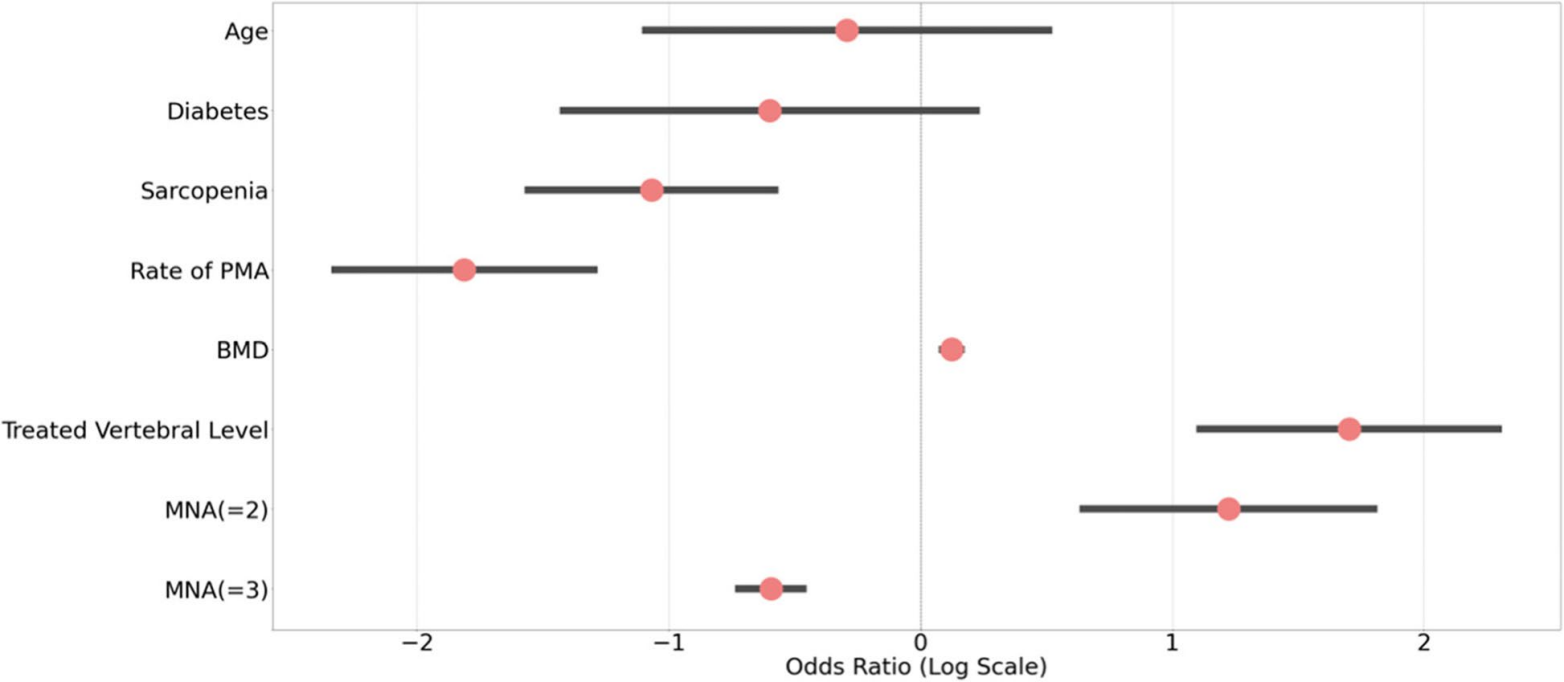
Multivariate analysis of factors associated with OVCRF

Table 2 shows the risk factors for OVCRF. Univariate analysis revealed a correlation between OVCRF and BMD, BMI, diabetes, sarcopenia, and age. Multivariate analysis showed that fatty infiltration of the PVM (OR: 1.13; CI: 0.02–0.22; *p* = 0.015), bone density (OR: 0.16; CI: -2.85–0.77; *p* = 0.001), sarcopenia (OR: 5.47; CI 0.51–2.89; *p* = 0.005), diabetes (OR: 3.39; CI: 0.07–2.38; *p* = 0.038), BMI (OR: 0.55; CI: -0.87–0.32; *p* < 0.001), and treated vertebral level (OR: 0.34; CI: -2.06–0.08; *p* = 0.034) remained as the independent predictors of OVCRF (Table 3, Fig. 4).

**Table 2:** Univariate analysis of factors associated with OVCRF

Variable	OVCRF (*n* = 74)	Non-OVCRF (*n* = 140)	*p*-value
Sex			
Male	20% (15)	32% (45)	0.066
Female	80% (59)	68% (95)	
Age (years)	80.97 ± 4.51	76.39 ± 5.4	< 0.001
Height (m)	1.56 ± 0.04	1.57 ± 0.05	0.1
Weight (kg)	46.45 ± 3.54	49.95 ± 4.31	< 0.001
BMI (kg/m^2^)	19.05 ± 1.25	20.17 ± 1.25	< 0.001
MNA			
A	4% (3)	18% (25)	< 0.001
B	28% (21)	47% (66)	
C	68% (50)	35% (49)	
BMD (T-score)	-2.99 ± 0.45	-2.18 ± 0.44	< 0.001
Diabetes			
Yes	42% (31)	10% (14)	< 0.001
No	58% (43)	90% (126)	
Sarcopenia			
Yes	78% (58)	8% (11)	< 0.001
No	22% (16)	92% (129)	
Treated vertebral level			
TL junction	55% (41)	61% (86)	0.394
Non-TL junction	45% (33)	39% (54)	
AMI (kg/m^2^)	5.25 ± 0.88	6.73 ± 1.07	< 0.001
Handgrip strength (kg)	11.68 ± 1.25	12.57 ± 1.21	< 0.001
Rate of PMA (%)	56.05 ± 4.36	48.9 ± 4.21	< 0.001

**Table 3:** Multivariate analysis of factors associated with OVCRF

	**Log odds**	**OR**	**95% CI**		***p*-value**
BMI	-0.59	0.55	-0.87	-0.32	< 0.001
Diabetes	1.22	3.39	0.07	2.38	0.038
Sarcopenia	1.7	5.47	0.51	2.89	0.005
Rate of PMA	0.12	1.13	0.02	0.22	0.015
BMD	-1.81	0.16	-2.85	-0.77	0.001
Treated Vertebral level	-1.07	0.34	-2.06	-0.08	0.034
MNA = 2	-0.6	0.55	-2.23	1.04	0.473
MNA = 3	-0.29	0.75	-1.89	1.31	0.72

## Discussion

OVCF is very common in the elderly population. In 2006, it was estimated that there are 1.4 million new fractures worldwide every year, and this number annually increases [25]. PKP is a minimally invasive technique for the treatment of painful OVCF. However, OVCRF is a serious complication of PKP in patients with osteoporotic vertebral fractures. Patients with OVCRF often experience severe back pain, which is difficult to control; therefore, a second surgical treatment may be required, which consequently increases the incidence of surgical complications. Moreover, the occurrence of OVCRF may also lead to secondary spinal stenosis, intervertebral disc disease, and spinal deformity, which may cause severe disability and poor quality of life [26]. Our study showed that OVCRF occurred in 64 (29.9%) of the 214 enrolled patients, which is similar to that reported in previous studies [27, 28].

A previous study summarized the possible risk factors for OVCRF and reported that advanced age, female sex, and low estradiol (E2) concentrations might be associated with post-PKP vertebral refracture [29]. According to Baek et al., the most important factors for VCRF are the degree of osteoporosis and altered biomechanics due to spinopelvic imbalance in the fractured area of the spine [30]. Lin et al. explained that significant anterior vertebral height restoration increases the risk of OVCRF [31]. Some studies have also focused on the impact of surgery-related factors such as bone cement leakage into the adjacent disc space [32]. Baroud et al. reported a *pillar-like* effect, wherein vertebral bodies augmented with bone cement become at least 12 times stiffer and 35 times stronger than control levels, resulting in increased pressure on adjacent vertebral levels [33]. In our study, we identified age, BMI, nutritional status, BMD, diabetes, and sarcopenia as significant risk factors for OVCRF. Multivariate analysis suggested that fatty infiltration of the PVM, BMD, sarcopenia, diabetes, BMI, and treated vertebral level remained as the independent predictors of OVCRF.

Rosenberg coined the term “sarcopenia” in 1989 to explain the decrease in muscle mass with age [34]. Muscle mass and muscle function begin to decline at the age of 35 years, and muscle strength declines faster than muscle mass, which is closely related to the results of our study. With the deepening of research, scholars have found that there are close mechanical effects between the adjacent interfaces of bone and muscle, so bone and muscle are increasingly considered as interacting tissues. Growing evidence suggests that several similar pathological pathways play an important role in sarcopenia and osteoporosis, including sensitivity to reduced anabolic hormone secretion, increased inflammatory cytokine activity, release of anabolic or catabolic molecules by the skeletal muscle or bone cells (i.e., myokines and osteokines), and reduced physical activity [35–37]. Osteoporosis increases the risk of fragility fracture in the elderly. However, previous studies have also shown that the prevalence of sarcopenia is higher in patients with fractures than in controls [38]. In 2009, Binkley and Buehring coined the terms *sarco-osteopenia* and *sarco-osteoporosis* to emphasize the weak bones and muscles that may contribute to falls and fractures among elderly adults [39]. Therefore, we propose that sarcopenia/PVM may also be risk factors for vertebral fracture recurrence. Therefore, considering the interference of BMD, we used multivariable analysis to process the data. After adjusting for these factors, sarcopenia remained a risk factor for OVCRF.

The PVM play an important role in the body’s trunk muscles; they maintain the stability of the spine and help with the movement of the vertebrae. Without muscular support, the spine has a compression threshold of only 2 kg before buckling [40]. Previous studies have pointed out that PVM are involved in the pathological process of spinal-related diseases, such as lower back pain and degenerative adult spinal deformity [41, 42]. Sarcopenia is an age-related disease that affects the skeletal muscles of the whole body. As the disease progresses, it is often accompanied by the deterioration of muscle quantity and quality throughout the body, leading to impaired balance and a high risk of fall-related injuries. Sarcopenia also leads to atrophy and degeneration of the lower back muscles, which decreases the stability of the lower back and increases the pressure of the vertebral body. PMA often manifests as fat infiltration. A previous study showed that a reduction in PVM mass (increased fat infiltration) is associated with postmenopausal osteoporotic vertebral fractures [43]. In our study, the mean (SD) atrophy rate in the OVCRF and non-OVCRF groups was 56.05% (± 4.36) and 48.9% (± 4.21), respectively (*p* < 0.001).

The multivariable analysis also showed that the atrophy rate was related to the OVCRF. After OVCF, a brace is often used to protect and maintain the stability of the spine. The lower back muscles play a role to some extent by protecting the spine from excessive flexion and reducing the load on the vertebral body. Patients with PMA lack protection of the PVM, which may result in OVCRF.

Clinically, we may need to pay more attention to the important role of muscles involved in OVCRF. Several studies have confirmed that even after timely surgery for OVCF, the lack of reasonable rehabilitation exercises, being bedridden for long periods, and using braces for a long duration can cause PMA [44], which is more serious in patients with sarcopenia and OVCF. Therefore, the atrophy process of the PVM should be prevented as much as possible. Katzman et al. also found that regular exercise significantly increased bone density and improved the blood supply and microcirculation of the spine vertebral body, thus increasing its strength [45]. Functional muscle exercises under the guidance of rehabilitation physicians can considerably reduce the incidence of OVCRF by inhibiting bone absorption and promoting bone formation.

## Conclusions

OVCRF has several risk factors. Sarcopenia is closely associated with osteoporosis. This study suggests that sarcopenia and PVM are independent risk factors for OVCRF. Therefore, to reduce the risk of OVCRF, strengthening the PVM after OVCF will reduce the risk of sarcopenia. This can be achieved through (i) discontinuation of spinal support use as soon as pain subsides, (ii) systematic functional muscle exercise to promote muscle contraction and balanced spine stress distribution, and (iii) adequate intake of nutrients, including protein or amino acid nutritional supplements, and active vitamin D.

## Supplementary Information

**Additional file 1.** 

**Additional file 2.** 

## Data Availability

All data generated or analysed during this study are included in this published article [and its supplementary information files].

## Abbreviations

AIC: Akaike information criterion
AMI: Appendicular muscle mass index
AWGS: Asian Working Group for Sarcopenia
BMD: Bone mineral density
BMI: Body mass index
CI: Confidence interval
DEXA: Dual-energy X-ray absorptiometry
MNA: Mini Nutritional Assessment
MRI: Magnetic resonance imaging
OR: Odds ratio
OVCF: Osteoporotic vertebral compression fracture
OVCRF: Osteoporotic vertebral compression refracture
PCSA: Physiological cross-sectional area
PKP: Percutaneous kyphoplasty
PMA: Paravertebral muscle atrophy
PVM: Paravertebral muscles
PVP: Percutaneous vertebroplasty
