# Supplementary material for: Relationship between sarcopenia/paravertebral muscles and the incidence of vertebral refractures following percutaneous kyphoplasty: a retrospective study
Source: BMC Musculoskelet Disord. 2022 Sep 22;23:879. doi: 10.1186/s12891-022-05832-6 (PMC9494877; doi:10.1186/s12891-022-05832-6)
Supplement: Supplementary file 2 — Additional file 2. [file 12891_2022_5832_MOESM2_ESM.docx]

**Sup 1** Original data
